# An assessment of solvent residue contaminants related to cannabis-based products in the South African market

**DOI:** 10.1186/s42238-022-00130-3

**Published:** 2022-04-12

**Authors:** Hendrik Jacobus Viviers, Anél Petzer, Richard Gordon

**Affiliations:** 1 National Analytical Forensic Services (NAFS), 109 Sovereign Drive, R21 Corporate Park, Centurion, South Africa; 2 North West University, Potchefstroom Campus, 11 Hoffman Street, Potchefstroom, South Africa

**Keywords:** Residual solvents, Ethanol, Isopropanol, South Africa, Medicinal cannabis, Chromatography, GC-MS, Cannabis oil, Plant extracts

## Abstract

Organic solvents are used for manufacturing herbal medicines and can be detected as residues of such processing in the final products. It is important for the safety of consumers to control these solvent residues. South African cannabis-based product samples were analysed for solvent residue contaminants as classified by the United States Pharmacopeia (USP), chapter 467. The origin of these samples ranged anywhere from crude extract, product development samples, and market ready final products. Samples were submitted to a contract laboratory over a period of 2 years from 2019 to 2021. To date, no data of this kind exist in South Africa specifically relating to cannabis-based medicinal, recreational, or complementary products. A total of 279 samples were analysed in duplicate by full evaporation headspace gas-chromatography mass-spectrometry and the results are reported in an anonymised format. The results showed an alarming 37% sample solvent residue failure rate with respect to adherence to USP 467 specification. It is important to ensure regulation is enforced to control product quality. The South African public need to be educated about the risks associated with cannabis-based products.

## Background

It is imperative to subject herbal preparations, medicines, and recreational products to quality control. For the pharmaceutical industry as well as cannabis in general there are an abundance of control measures in place to ensure their safety and efficacy (WHO, 2007; Viviers et al., 2021). A range of organic solvents are used for manufacturing herbal medicines and can be detected as residues of such processing in the final products. Medicinal cannabis extracts and other processed forms may thus contain residual solvents. This is especially relevant to extracts which have a sticky and viscous nature that make it difficult to remove solvents (Romano & Hazekamp, 2013). The most common examples of such cannabis extracts are termed “Rick Simpson oils” or “FECO’s” (full extract cannabis oil).

Cannabinoids as well as terpenoids and flavonoids are extracted by a solvent, followed by an evaporation step in order to increase the concentration of these compounds in the extract (Romano & Hazekamp, 2013; Hazekamp, 2006). These types of cannabis oils or extracts are becoming increasingly popular amongst self-medicating patients because of the simplicity and low cost involved in producing the oils (Romano & Hazekamp, 2013). After solvent evaporation, residues are still present in the extract and the concentrations of the solvent residues should be controlled through good manufacturing practice (GMP) and quality control of the final products (International Community of Harmonization (ICH), 2011). To ascertain whether a product is safe for chronic human consumption, The International Council for Harmonisation of Technical Requirements for Pharmaceuticals for Human Use (ICH) as well as the United States Pharmacopoeia (USP) have listed predetermined solvent residue limits. Solvents are classified by ICH (International Community of Harmonization (ICH), 2011) as well as the USP (United States Pharmacopeia (USP), 2009), according to their potential risks into the following categories:

- Class 1 (Solvents to be avoided such as benzene which are potentially carcinogenic);
- Class 2 (Toxic potential such as methanol or acetonitrile);
- Class 3 (Limited toxic potential such as ethanol).

Residual solvents are primarily analysed by Headspace GC-FID (gas chromatography-flame ionization detection) or liquid injection GC-FID (United States Pharmacopeia (USP), 2009). The alternative use of MS (mass spectrometry) detection may provide additional selectivity for co-eluting solvents. In this study, a published analytical method was employed as basis for the development of the final analysis method for residual solvents (Hilliard et al., 2009). As a result of the viscosity of most of the cannabis extracts, liquid injection is not feasible. Although ICH and USP (International Community of Harmonization (ICH), 2011; United States Pharmacopeia (USP), 2009) provide a guideline that lists the solvents which should be included in a working list, the solvents that will be analysed are ultimately decided by each manufacturer or quality control laboratory.

From interaction with cannabis extract manufacturers Table 1 was compiled and provide a list of the solvents that have been analysed for each class. This list is by no means exhaustive and could be altered to include additional solvents. Few manufacturers employ class 1 and 2 solvents, nonetheless they are included.

**Table 1:** Residual solvent limits imposed by ICH and USP (International Community of Harmonization (ICH), 2011; United States Pharmacopeia (USP), 2009)

	Solvent		Limit (ppm)
Class 3	2-butanol		< 5000
	Acetone		< 5000
	Butanone/methyl ethyl ketone		< 5000
	Diethyl ether		< 5000
	Ethanol		< 5000
	Ethyl acetate		< 5000
	Isopropanol (IsoOH)		< 5000
	Methyl tertbutyl ether (MTBE)		< 5000
Class 2	1.4-Dioxane		< 380
	Acetonitrile		< 410
	Chlorobenzene		< 360
	Cyclohexane		< 3880
	Cumene/isopropyl benzene		< 70
	Methanol		< 3000
	Methyl cyclohexane		< 1180
	Methylene chloride		< 600
	Ethylbenzene	Total xylenes	< 2170
	o-xylene		
	p- and m-xylene		
	Tetrahydrofuran		< 720
	Toluene		< 890
	Trans 1,2-dichloroethene	Total dichloroethenes	< 1870
	cis-dichloroethene		
Class 1	Benzene		< 2

An evaluation of the quality of medicinal cannabis-based products has been performed previously (Hazekamp, 2006), but was only limited to samples in the Netherlands. This study included solvent residue testing but only to extend of comparing different production methods with one another using different types of solvent. It should be noted that due to the high viscosity of the extracts, significant solvent residues were also reported by this study (Romano & Hazekamp, 2013). As study evaluating the quality of artisanal/home produced cannabis-based products, used to manage seizures in children, was conducted in 2022 (Suraev et al., 2022). Of the 58 cannabis sample evaluated, 17 (29%) contained concentrations of ethanol and isopropanol above USP solvent residue limits (Suraev et al., 2022). A study conducted in 2015 found that isopentane was the most frequently detected (29.8%) solvent residue in 57 samples (Raber et al., 2015). Other residual solvents detected were butane, heptane, hexanes, isobutane, isopropanol, neopentane, pentane, and propane (Raber et al., 2015). It should be noted that this article only reported solvent residues detected and not solvent residues above a specified limit. With this background, the aim of this study was to analyse a segment of the South African cannabis-based products in circulation and to provide a detailed overview of the solvent residues contained in these products. Furthermore, the adherence of these samples to the imposed limits by the ICH and USP will also be evaluated (International Community of Harmonization (ICH), 2011; United States Pharmacopeia (USP), 2009). To date, no data of this kind exists in South Africa. The output of the study will provide insights to manufacturers, the public, and regulators alike.

## Materials and methods

An assortment of samples from a variety of manufacturers in South Africa were submitted to the contract laboratory. Manufacturers are defined as any type of user, retailer, reseller, producer, or importer of cannabis-based products. Whether these manufacturers maintain the full value chain or only a portion thereof they are defined as manufacturers for the purpose of this study. Manufacturers may include cultivators of plants, producers of products, importers, resellers, and pharmaceutical manufacturers. All samples were submitted to a contract laboratory for analysis and the data will be represented in an anonymised format. Solvents from classes 1, 2, and 3 were analysed (see Table 1). Depending on the solvents employed in the manufacturing process, the appropriate solvent class was chosen by each manufacturer for each specific sample submission. A screen of all available solvents would be impractical; thus, manufacturers selected the solvent class which was most likely present in the final product. In total, 299 samples were analysed in duplicate, a total of 598 datapoints. Consent was provided to employ the data for research purposes. The majority of samples submitted for residual solvent analysis could be defined as any sample prepared or processed in a way to extract cannabinoids from plant material to up-concentrate the cannabinoid content in the final product. These might include full extract cannabis oil (FECO), Rosin, Rick Simpson oil (RSO), Hashish, Butane hash oil (BHO), etc.

Obtained from Perkin Elmer Corporation (Waltham, MA, USA), a Clarus 680 gas chromatograph equipped with a SQ8 mass spectrometer and HS40 Headspace autosampler was employed for HS-GC-MS analysis. A Zebron ZB-624 (30 m, 0.32 mm × 1.8 μm) capillary column obtained from Phenomenex (Torrance, CA, USA) was employed for separation. Two reference methods were employed as a starting point and were altered to achieve more desirable run times (United States Pharmacopeia (USP), 2009; Hilliard et al., 2009). Sample preparation was conducted as described by Hilliard (Hilliard et al., 2009). Residual solvent certified reference materials (CRMs) were obtained from Sigma-Aldrich (St. Louis, MO, USA). The following headspace instrumental parameters were employed. Needle temperature 175 °C, vail oven temperature 170 °C, transfer line temperature 175 °C, GC cycle time 32 min, injection volume 20 μL, pressurise time 1.0 min, thermostat time 20 min, withdraw time 0.1 min. GC parameters included: Injection port temperature 220 °C, split ratio 5:1, carrier gas: (helium) flow 1.2 mL/min. The GC oven program initial temperature was set to 44 °C hold for 16 min, ramp 1–12 °C/min to 153 °C, ramp 2–50 °C /min to 230 °C hold for 1.5 min. The MS transfer line was set to 200 °C with the source set at 150 °C. Table 2 shows the MS m/z ions monitored.

**Table 2:** m/z MS ions monitored

	Solvent	m/z ions
Class 3	2-butanol	45, 59
	Acetone	42, 58
	Butanone/methyl ethyl ketone	43, 72
	Diethyl ether	45, 59
	Ethanol	31, 45
	Ethyl acetate	43, 61
	Isopropanol (IsoOH)	43, 45
	Methyl tertbutyl ether (MTBE)	57, 73
Class 2	1.4-dioxane	58, 88
	Acetonitrile	40, 41
	Chlorobenzene	77, 112
	Cyclohexane	56, 84
	Cumene/isopropyl benzene	105, 120
	Methanol	31, 32
	Methyl cyclohexane	55, 83
	Methylene chloride	49, 84
	Ethylbenzene	91, 106
	o-xylene	91, 106
	p- and m-xylene	91,106
	Tetrahydrofuran	42, 72
	Toluene	91, 92
	Trans 1,2-dichloroethene	61, 96
	cis-dichloroethene	61, 96
Class 1	Benzene	52, 78

A limit test procedure was employed for the analysis of all residual solvents. A calibration standard was prepared at the concentration limit of each individual solvent using the first batch of CRM. A one-point calibration was employed for each USP limit of each individual solvent. A second batch of CRMs was employed to prepare a control standard, again at the limit concentration stipulated by USP monograph 467 (United States Pharmacopeia (USP), 2009). Analysis commenced with a blank run, then a calibration standard, followed by a control standard every 10 injections, to avoid instrumental drift. Sample sets were concluded by a control standard to ensure all samples within a sample set adhered to bias and variation limits. Control standard specifications were 15% RSD at the concentration limit, and re-calibration was performed when control standards fell outside these specifications. This method was regarded as quantitative with a very narrow range of 15% across the USP 467 concentration limit. Any values falling outside this window were regarded as semi-quantitative only. Since manufacturers are only interested to know whether the products fall within a certain specified concentration limit, a full quantitative analysis was redundant.

The data was subdivided into three different categories:

1. Individual solvent analytes were grouped together with no relationship to the sample. This was done to determine the number of times this specific solvent failed to adhere to the USP/ICH (International Community of Harmonization (ICH), 2011; United States Pharmacopeia (USP), 2009) specification. Furthermore, the number of times a certain solvent was present, irrespective of adherence to a specification limit, was also determined and noted as either present when it occurred or not detected (ND).
2. Secondly, samples were grouped together to determine the frequency of sample failures as well as the frequency by which the test panel solvents are present in a specific sample. The frequency of failed samples only considered whether any one of the solvents in the test panel failed.
3. Lastly with the samples grouped together, the total amount of solvent detected in the sample was summed together and compared to a set adherence limit. This manner, even if individual solvents within a sample adhered to the USP specification limit, the sum total concentration of solvent present in a sample may exceed the set adherence limit.

## Results

The results of the analyses are shown in Appendix, Table 3. The results are grouped according to individual solvent analytes and shows the occurrence of each individual solvent across the 298 samples. In Appendix, Table 4 shows sample failures as well as whether a solvent was present in the sample irrespective of the safety limit. In Appendix, Table 5 shows sample failures when summed solvents concentrations are considered against a safety limit.

**Fig. 1:**
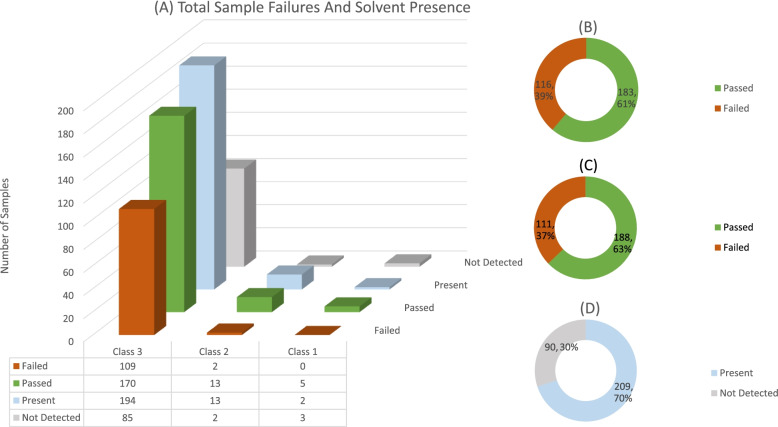
Number of sample failures, and the number of samples that contained solvent (**A**). Total percentage of sample failures for all classes, if all solvents present in a sample are summed (**B**). Total number of sample failures for all classes where one solvent exceeded the limit (**C**). Total percentage of samples for all classes where any solvent was detected in sample (**D**)

**Fig. 2:**
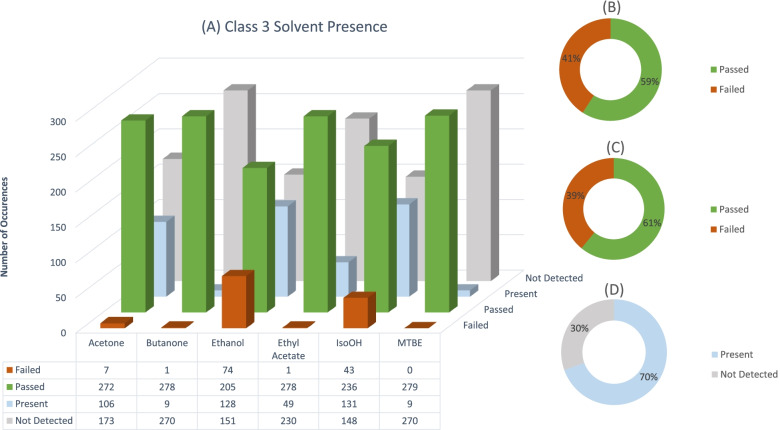
Number of Individual solvent failure occurrences, and the number of times a class 3 solvent was present (**A**)..Total percentage of sample failures for class 3 solvents, if all solvents present in a sample are summed (**B**). Total number of sample failures for all class 3 solvents where one solvent exceeded the limit (**C**). Total percentage of samples for class 3 solvents where any solvent was detected in sample (**D**)

**Fig. 3:**
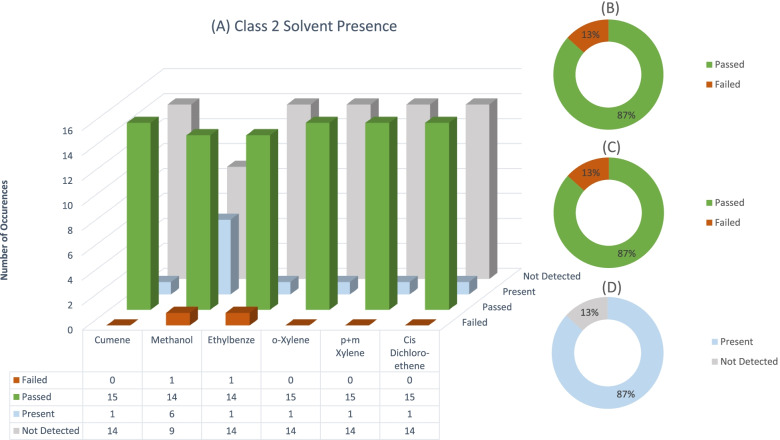
Number of Individual solvent failure occurrences, and the number of times a class 2 solvent was present (**A**). Total percentage of sample failures for class 2 solvents, if all solvents present in a sample are summed (**B**). Total number of sample failures for all class 2 solvents where one solvent exceeded the limit (**C**). Total percentage of samples for class 2 solvents where any solvent was detected in sample (**D**)

Figure 1 shows the individual solvent failures as well the number of times each solvent occurred or was detected for all three solvent classes. Since most of samples that were analysed contain class 3 solvents, Figs. 2 and 3 illustrate the number of failures as well as the occurrence of each individual solvent in class 2 and class 3. It should be noted that for Figs. 2 and 3, only solvents which were present are displayed. Additionally, Fig. 1, 2, and 3 pie graphs B, C, and D show the amount of sample failures, the amount of sample failures if concentrations are summed, and finally the overall occurrence/detected solvents in all samples.

## Discussion

The data represents an overview of cannabis-based products in South Africa that were screened for solvent residues. This data should not be regarded as a representation of the entire South African cannabis market since not every product on the market was analysed. Each solvent class will be discussed individually starting with the solvents that were present in most samples. Solvents that were not detected in any of the samples but were included in the test panel will be omitted from the figures.

### Class 3

Class 3 solvents occurred in most of the samples. Solvent residues in this class have the least stringent specification limits (International Community of Harmonization (ICH), 2011; United States Pharmacopeia (USP), 2009). This can be viewed in a positive light since most samples analysed fell within this class and can be considered the least dangerous. When considering individual solvent analytes, the most prevalent solvent residue failure was ethanol, followed by isopropanol and acetone (see Fig. 2). When considering the presence of these residues in samples, ethyl acetate should also be mentioned. Although ethyl acetate had only 1 residue failure, it was present in almost 50 samples. Additionally, although isopropanol had less failures than ethanol, isopropanol residues were present in more samples than ethanol. A reason for the prevalence of these solvent residues might be the ease of acquiring such solvents from a variety of vendors in South Africa. A variety of manufacturers also do not employ food grade or high purity ethanol, which contains denaturants like ethyl acetate and acetone, since high purity ethanol is taxed at a significant rate in South Africa. Comparing individual class 3 solvent residue failures to summed solvent residue failures, summed solvent residue failures only increased by a small fraction (39% failed individual vs 41% failed summed). It could be argued then that most manufacturers employ a single moderately pure solvent in their production process. A high percentage of class 3 samples (69.5%) contained detectable concentrations of solvent residues even though these residues were not above the specification limit.

### Class 2

Class 2 solvents were second most prevalent in the sample dataset. Solvent residues in this class have more stringent specification limits (International Community of Harmonization (ICH), 2011; United States Pharmacopeia (USP), 2009). These solvents should be viewed with concern by home producers as they are more dangerous to human health. Considering the individual class 2 solvents, only 1 failure occurred for each of methanol and ethylbenzene (see Fig. 3), yielding a 15% failure rate. A high percentage of class 2 samples (87%) contained detectable concentrations of solvent residues even though these residues were not above the specification limit. Although these solvents pose higher health risks, the lower failure rate of class 2 solvents is a positive finding since less manufacturers employ these solvents. What should be noted is the much higher percentage of class 2 solvents that are present in final products submitted for this test panel. This might translate to class 3 solvents being easier to remove during manufacturing than class 2 solvents. Alternatively, because of the lower detection limits needed to detect class 2 solvents compared to class 3 solvents, they are detected at a higher frequency in samples.

### Class 1

Class 1 solvents occurred the least in the samples. Solvent residues in this class have the most stringent specification limits, and should be avoided (International Community of Harmonization (ICH), 2011; United States Pharmacopeia (USP), 2009). These solvents are also recognised as carcinogens (Baker, 1994). The samples were only screened for the presence of benzene in this category and a 0% failure rate occurred, even with an imposed specification limit of 2 ppm (Appendix, Table 3 and Table 4). Benzene was present in only two samples, and it should be noted that in both instances benzene concentrations were below the specified 2 ppm USP/ICH limit (International Community of Harmonization (ICH), 2011; United States Pharmacopeia (USP), 2009).

The data of all solvent classes were pooled as shown in Fig. 3, which indicates the total number of samples submitted for any class solvent residue analysis that failed. Among the 279 samples, a total of 111 samples had at least one solvent analyte that failed the USP/ICH specification limit (37% failure rate). A study conducted on solvent contaminants in cannabis-based products employed to treat epilepsy, averaged a USP specification failure rate of 29% (Suraev et al., 2022), compared to the significantly higher South African failure rate of 37%.

For summed solvent concentrations the failure rate increased by a marginal 2%, which could be interpreted to mean that manufacturers use a single moderately pure solvent during their production. This is supported by the finding that only high failing concentrations of a single solvent are usually present in a specific sample. The overall presence of any solvent in a sample is displayed in Fig. 3. A high percentage of samples (70%) contained detectable concentrations of solvent residues even though these residues might not have been above the specification limit.

Another study conducted in 2015 found solvent detection percentages of 71.9% (Raber et al., 2015), comparable to the South African 70%. It should also be noted that not all samples submitted employed solvent extraction as means of manufacturing. A limited number of samples were submitted to obtain a Certificate of Analysis which shows compliance to USP 467 or ICH Q3C, even though no solvent was used in production.

## Conclusion

In conclusion, when assessing solvent residues present in samples against a pharmacopeial safety limits, it is evident that a large fraction of cannabis-based products in South Africa exceeds these limits. These findings are either comparable or show higher failure rates than other studies published (Suraev et al., 2022; Raber et al., 2015). Even though safety limits for solvent residues have been published, adherence by manufacturers is lacking, notwithstanding enforcement of these limits by regulators. With a 37% overall sample solvent residue failure rate, it is alarming that these products are being distributed in South Africa. It is the aim with the publication of this data to inform the public and regulators alike.

## Data Availability

The datasets generated and/or analysed during the current study are not publicly available due to the data being anonymised contract laboratory samples, the summarised data is available from the corresponding author at henrick@nafs.co.za on reasonable request. It should be noted however that either certain sample details will be redacted, or not be available. Raw data reports are not available.

## Appendix

Tables 3, 4 and 5

**Table 3:** Individual solvent failures and occurrence in samples

			USP limit (ppm^a^)	Passed	Failed	Present	Not detected
Class 3	**2-butanol**		< 5000	279	0	0	279
	**Acetone**		< 5000	272	7	106	173
	**Butanone**		< 5000	278	1	9	270
	**Diethyl ether**		< 5000	279	0	0	279
	**Ethanol**		< 5000	205	74	128	151
	**Ethyl acetate**		< 5000	278	1	49	230
	**IsoOH (isopropanol)**		< 5000	236	43	131	148
	**MTBE (tertbutyl methyl ether)**		< 5000	279	0	9	270
Class 2	**1.4 dioxane**		< 380	15	0	0	15
	**Acetonitrile**		< 410	15	0	0	15
	**Chlorobenzene**		< 360	15	0	0	15
	**Cyclohexane**		< 3880	15	0	0	15
	**Cumene (isopropyl benzene)**		< 70	15	0	1	14
	**Methanol**		< 3000	14	1	6	9
	**Methyl cyclohexane**		< 1180	15	0	0	15
	**Methylene chloride**		< 600	15	0	0	15
	**Ethylbenze**	**Total xylenes**	< 2170	14	1	1	14
	**o-xylene**			15	0	1	14
	**p+m xylene**			15	0	1	14
	**Tetrahydrofuran**		< 720	15	0	0	15
	**Toluene**		< 890	15	0	0	15
	**Trans 1,2 dichloroethene**	**Total dichloroethenes**	< 1870	15	0	0	15
	**Cis dichloro-ethene**			15	0	1	14
Class 1	**Benzene**		< 2	5	0	2	3
			***Total***	***2334***	***128***	***445***	***2017***

^a^*ppm* parts per million

**Table 4:** Number of sample failures and the occurrence of solvents in samples

			USP limit (ppm^a^)	Passed	Failed	Present	Not detected
Class 3	**2-butanol**		< 5000	170	109	194	85
	**Acetone**		< 5000				
	**Butanone**		< 5000				
	**Diethyl ether**		< 5000				
	**Ethanol**		< 5000				
	**Ethyl acetate**		< 5000				
	**IsoOH (Isopropanol)**		< 5000				
	**MTBE (tertbutyl methyl ether)**		< 5000				
Class 2	**1.4 Dioxane**		< 380	13	2	13	2
	**Acetonitrile**		< 410				
	**Chlorobenzene**		< 360				
	**Cyclohexane**		< 3880				
	**Cumene (isopropyl benzene)**		< 70				
	**Methanol**		< 3000				
	**Methyl cyclohexane**		< 1180				
	**Methylene Chloride**		< 600				
	**Ethylbenze**	**Total xylenes**	< 2170				
	**o-xylene**						
	**p+m xylene**						
	**Tetrahydrofuran**		< 720				
	**Toluene**		< 890				
	**Trans 1,2 Dichloroethene**	**Total dichloroethenes**	< 1870				
	**Cis dichloro-ethene**						
Class 1	**Benzene**		< 2	5	0	2	3
			***Total***	***188***	***111***	***209***	***90***

^a^*ppm* parts per million

**Table 5:** Sample failures when solvent concentrations are summed

			USP limit (ppm^a^)	Passed	Failed
Class 3	**2-butanol**		< 5000	165	114
	**Acetone**		< 5000		
	**Butanone**		< 5000		
	**Diethyl ether**		< 5000		
	**Ethanol**		< 5000		
	**Ethyl acetate**		< 5000		
	**IsoOH (isopropanol)**		< 5000		
	**MTBE (tertbutyl methyl ether)**		< 5000		
Class 2	**1.4 dioxane**		< 380	13	2
	**Acetonitrile**		< 410		
	**Chlorobenzene**		< 360		
	**Cyclohexane**		< 3880		
	**Cumene (isopropyl benzene)**		< 70		
	**Methanol**		< 3000		
	**Methyl cyclohexane**		< 1180		
	**Methylene chloride**		< 600		
	**Ethylbenze**	**Total xylenes**	< 2170		
	**o-xylene**				
	**p+m xylene**				
	**Tetrahydrofuran**		< 720		
	**Toluene**		< 890		
	**Trans 1,2 dichloroethene**	**Total dichloroethenes**	< 1870		
	**Cis dichloro-ethene**				
Class 1	**Benzene**		< 2	5	0
			***Total***	***183***	***116***

^a^*ppm* parts per million

## Abbreviations

BHO: Butane hash oil
CRM: Certified reference material
FECO: Full extract cannabis oil
GC-FID: Gas chromatography-flame ionization detection
GMP: Good manufacturing practice
ICH: The International Council for Harmonisation of Technical Requirements for Pharmaceuticals for Human Use
MS: Mass spectrometry
ND: Not detected
RSD: Relative standard deviation
RSO: Rick Simpson oil
USP: United States Pharmacopeia
